# Investigating routine care non-pharmacological treatment for adolescents with ADHD

**DOI:** 10.3389/frhs.2022.929521

**Published:** 2022-08-17

**Authors:** Margaret H. Sibley, Juan Carlos Reyes Francisco, Alexandria Rios-Davis, Paulo A. Graziano

**Affiliations:** ^1^Department of Psychiatry and Behavioral Sciences, University of Washington School of Medicine, Seattle, WA, United States; ^2^Center for Child Health, Behavior, and Development, Seattle Children's Research Institute, Seattle, WA, United States; ^3^Department of Psychology, Florida International University, Miami, FL, United States

**Keywords:** Attention Deficit and Hyperactivity Disorder (ADHD), adolescent, community mental health, behavior therapy, mixed method

## Abstract

**Objective:**

To characterize routine non-pharmacological care for youth with ADHD.

**Methods:**

76 audio-recorded work-samples were collected from community mental health therapists in a large metropolitan area in the United States and were analyzed for operationally defined practice elements commonly included in evidence-based non-pharmacological treatment for ADHD. Analyses characterized community provider practices and examined predictors of using evidence-based (vs.low-value) practices.

**Results:**

Individually delivered social skills training was the most commonly detected practice element (31.6% of practice samples). Parent involvement in routine care was uncommon (53.9% of sessions had no parental presence). Core elements of evidence-based practices were rarely delivered (e.g., organization skills training: 18.4% of tapes; operant reinforcement: 13.2%); when evidence-based content was introduced, it was typically implemented at a very low intensity. Patient and provider characteristics did not predict use of evidence-based practices.

**Conclusions:**

Routine non-pharmacological care for adolescent ADHD primarily consisted of low value practices such as youth-directed treatment and social skills training with low parent involvement and only occasional therapy homework. To improve quality of care, efforts to de-implement low value practices should be coupled with efforts to implement evidence-based practices (i.e., parent involvement, measurement-based care, organization skills training, use of operant reinforcement).

## Introduction

Adolescence is a critical period for ADHD treatment given close relationships between adolescent functioning and adult outcomes (1–3). The two evidence-based treatments for ADHD are medication (stimulant or non-stimulant) and (cognitive) behavioral treatments (psychosocial treatment (4–6). Though routine care medication practices have been characterized elsewhere (7), there is almost no information on routine care psychosocial treatment for ADHD, including in adolescence.

While psychosocial treatments for children with ADHD typically are limited to training parents to apply operant behavioral strategies (4), adolescent treatments incorporate cognitive and skills-based components delivered directly to the adolescent (8). Outcome data for adolescent ADHD psychosocial treatments are robust (8–12). Unlike childhood behavioral treatments for ADHD (13), adolescent treatments may demonstrate long-term effects (14). ADHD remains the most common diagnosis in youth mental health clinics (15, 16) and service delivery for childhood externalizing disorders (including ADHD) suggests that evidence-based interventions are rarely implemented in favor of low-value practices (e.g., play therapy) (17). However, no work has examined the implementation of evidence-based psychosocial interventions for ADHD in adolescence. Investigating routine psychosocial care for adolescents with ADHD is a first step toward identifying practice needs and promoting evidence-based non-pharmacological care.

The present study analyzes 76 work samples submitted by 26 community therapists at four mental health clinics who delivered behavior therapy to 74 adolescents with a primary diagnosis of ADHD. Work samples were collected as part of a routine care condition in a randomized community-based effectiveness trial of an ADHD behavior therapy (18). Herein, we comprehensively characterize the presence of evidence-based (vs. low value) practice elements. We report the presence and extensiveness of each identified practice element, who attended sessions, and whether implementation of evidence-based practice elements was associated with therapist or youth characteristics, as well as engagement and treatment outcomes. We hypothesized that therapists would primarily use low-value practices in favor evidence-based approaches; however, due to a lack of research on routine psychosocial care for ADHD, we did not have hypotheses about specific low value practices that might be used.

## Methods

### Study design

Full study design and CONSORT diagram are described elsewhere (18). Herein we analyze 76 work samples provided by 26 community mental health therapists who treated 74 adolescent participants as a part of the routine care control group of a randomized community-based trial of adolescent ADHD treatment. There was double randomization of therapists and adolescents to the evidence-based intervention group or routine care. The sample included 111 adolescents with ADHD who received routine care therapy for ADHD; however, 37 did not provide usable audio recordings.

### Participants

#### Adolescents

The 74 adolescents (ages 11–17) received routine mental health therapy for ADHD at one of four community mental health clinics in a large U.S. city. Participants were required to meet DSM-5 ADHD criteria according to a systematic evaluation conducted by the research team that integrated parent and teacher ratings of symptoms and impairment. Autism spectrum disorder and intellectual disability (IQ < 70) were exclusionary. Full characteristics of the subsample can be found in Table 1. Average age of the sample was 14.34 years (*SD* = 1.50). The sample 62.6% male, 90.8% Latinx, 6.8% Black or African-American, and represented a broad socio-economic distribution. Approximately 20% of the sample received naturalistic adjunctive stimulant medication for ADHD during psychosocial treatment.

**Table 1:** Baseline characteristics of adolescent subsample (*N* = 74).

**Diagnostic variables**	
WASI estimated Full-Scale IQ *M* (*SD*)	97.97 (11.92)
**ADHD subtype**	
ADHD-predominantly inattentive (%)	54.1
ADHD-combined (%)	45.9
ODD/CD (%)	48.6
Current ADHD medication (%)	20.3
**Demographic variables**	
Age *M*(*SD*)	14.34 (1.50)
Male (%)	62.2
Race/ethnicity (%)	
White non-hispanic	1.4
Black non-hispanic	6.8
Hispanic any race	90.5
Other	1.3
Single parent (%)	32.4
Parent limited english proficiency (%)	59.5
**Parent education level**	
High school grad, GED, or less (%)	24.7
Part college or specialized training (%)	28.8
College or university grad (%)	38.4
Graduate professional training (%)	8.1

WASI, Wechsler Abbreviated Scale of Intelligence; ODD, Oppositional Defiant Disorder; IQ, Intelligence Quotient; ADHD, Attention Deficit Hyperactivity Disorder; *M*, Mean, *SD*, Standard Deviation; CD, Conduct Disorder; GED, General Educational Diploma.

Two adolescent participants transferred therapists during the course of treatment and, therefore, were represented on two separate work samples submitted by separate therapists. Usual care participants represented on work samples were more likely to have a parent with limited English proficiency (*p* < 0.001) and were slightly older (*p* = 0.032) than those not represented on work samples. They also attended significantly more sessions than those without a work sample (*p* < 0.001; likely due to adolescents discontinuing treatment prior to a work sample being recorded).

#### Therapists

Therapists (*N* = 26) were mental health professionals employed at four community agencies. Therapists self-identified as 15.4% non-Hispanic White, 15.4% Black or African-American, and 65.4% Hispanic. They were 80.8% female therapists, with 65.4% offering treatment in both Spanish and English. 19.2% of therapists were licensed and 84.6% held a master's degree (7.7% held a doctorate and 7.7% were bachelor's level). On average, clinicians reported 6.73 years of experience delivering therapy (range: 1–31).

There were no demographic differences between usual care therapists who were represented on work samples (*n* = 26) and those who were not (*n* = 14). Reasons for missing work samples included therapist refusal, inaudible recordings, parents who did not consent to this aspect of the research, participants who dropped out of treatment before work samples could be collected, and human error.

### Procedures

#### Treatment delivery

Interventions were provided by agency employees using typical billing procedures. Therapists in the routine care group were asked to provide one audio-recorded work sample per study case. Therapists were instructed to treat study cases using usual procedures in the agency and the treatments they believed would be most effective for the youth. They received supervision for their study cases from agency supervisors according to typical agency practices. Therapists were not given access to any study intervention materials and no contamination with the active treatment group was found (18).

### Measures

#### Adolescent ADHD treatment elements rating system

The AATERs includes 18 distinct evidence-based practice element codes for adolescent ADHD treatment (19). The AATERS manual contains in depth definitions of each code, common, rare, and difficult examples, decision-rules, tips for analyzing therapist behaviors, and contrasting examples. Using coding procedures adapted from the Therapy Process Observational Coding System for Child Psychotherapy Strategies Scale (TPOCS) (20), coders segmented each recording into 5-min intervals and separately coded the presence of each AATERS code in each interval. At the conclusion of the full recording, coders applied a 1 (not at all) to 7 (extensively) extensiveness rating for each code based on the thoroughness and frequency at which codes appeared on the tape. Coders also indicated who attended the session, the percentage of the session each attendee was present, and language of the session (English or Spanish). Twenty percent of tapes (15 tapes) were randomly selected for inter-rater reliability coding by two trained research coders, which was strong: intraclass correlations (ICCs) ranged from 0.63 to 0.92^1^, with an average ICC of 0.79, indicating good inter-rater reliability.

#### Predictors of EBP utilization

Parents and therapists completed demographic questionnaires at the beginning of the study. Family adversity using an existing adaptation of the Rutter Family Adversity Index (21, 22), which was modified to fit the sample context (i.e., high prevalence of immigrant families) and available data. The resulting score (0–4) equally weighed the following risk factors: (1) single parent household, (2) all parents with a high school degree or less (indicator of low socio-economic status), (3) all parents with limited English proficiency, and (4) greater than two children living in the home. Classification variables in the latent profile analysis (LPA) included: teacher reports of disruptive classroom behavior and organization, time management, and planning (OTP) problems, youth report of anxiety and depression, full-scale IQ scores, ADHD subtypes assessed by structured parent interview and teacher symptom ratings at clinical intake, Oppositional Defiant Disorder (ODD) and Conduct Disorder (CD) symptoms reported by the parent for the home setting, and school records of academic impairment. For profile and measurement details, see Coxe et al. (21). The “ADHD simplex” profile was characterized by a mix of the ADHD-IN and ADHD-Combined subtypes, moderate impairment levels, and infrequent comorbidities. “ADHD+internalizing” was characterized by higher likelihood of clinically elevated comorbid anxiety and/or depression. The “disruptive/disorganized ADHD” profile was characterized by severe OTP problems, ADHD-C subtype, slightly lower IQ, and frequent disruptive behavior at school. Higher scores indicated more risk factors.

### Analytic plan

We calculated the prevalence of each EBP element across the 76 work samples, the average extensiveness of each EBP element when implemented, the average number of EBP elements present per session, and the percentage of sessions with at least one EBP element.

Based on guidelines from the TPOCS (20), we categorized sessions as having “high” EBP implementation if they had at least one practice element scored as a “5 or higher” on the AATER extensiveness scale (i.e., “considerably to extensively”). We categorized “low” EBP implementation if they had at least one EBP present, but no EBPs rated above a “4” on the extensiveness scale (i.e., “somewhat”). We categorized “no” EBP implementation if they had no EBPs implemented during the session. Multinomial logistic regression analyzed three EBP implementation groups as unordered categories with “low” EBP implementation as the reference group. Service-level predictors included: therapist licensure status, therapist years of experience, language of treatment, and whether the parent was present. Patient-level predictors were: family adversity, two presenting problems dummy codes with ADHD simplex as reference group, and age.

## Results

### Session characteristics

The adolescent was present for the full session in 100% of samples. A parent was present in 46.1% of the sample sessions (*n* = 35); in 62.9% (22 of 35) of these, the parent attended at least 75% of the session. Thus, 28.9% of sessions were considered joint parent-teen sessions, 17.1% were adolescent-directed with some parent involvement, and 53.9% were adolescent-only sessions. Four sessions (5.3%) included another family member such as a second parent or sibling. 30.3% of sessions were conducted in Spanish.

### Practice elements

81.6% of work samples included at least one practice element. On average, sessions integrated 2.14 practice elements per session (*SD* = 1.65). Table 2 shows the prevalence of each element (range: 0.0–31.6%). The most frequent elements were social skills training (31.6%), skill application homework (28.9%), and time management/planning training (22.4%).

**Table 2:** Prevalence and extensiveness of adolescent ADHD EBP elements on 76 samples.

**Practice element**	**Prevalence (%)**	**Extensiveness *M(SD)***
Any EBP present in session	81.6	–
Social skills training	31.6	3.04 (1.33)
Skill application assignments	28.9	2.68 (1.04)
Time management/planning training	22.4	2.47 (0.72)
Organization skills training	18.4	2.29 (0.47)
Cognitive restructuring	17.1	2.85 (1.07)
Parent-teen communication training	15.8	2.83 (1.04)
Distractibility reduction training	14.5	2.18 (0.40)
Behavioral parent training	13.2	3.00 (1.63)
Progress monitoring	13.2	2.40 (0.70)
Rewards systems	13.2	2.90 (1.52)
Parent-delivered	9.2	3.29 (1.70)
In-session	2.6	2.00 (0.00)
Psychoeducation on ADHD	11.8	2.56 (1.01)
Academic skills training	5.3	3.25 (0.96)
Assessment feedback	3.9	2.00 (0.00)
Personal goal setting	3.9	2.67 (0.58)
Structured problem-solving	1.3	2.00 (0.00)
Self-monitoring training	1.3	2.00 (0.00)
Direct teacher engagement	0.0	–
Peer recreational activities	0.0	–

When an element was delivered, its average extensiveness ranged from 2.00 to 3.29 on the 1–7 extensiveness scale (see Table 2). Social skills training, academic skills training, parent-delivered rewards, and behavioral parent training had the highest extensiveness (3.00–3.29). At best, practice elements were implemented at an average of “3-somewhat.”

### Predictors of EBP implementation

Sessions were classified as 11.8% high EBP implementation, 69.7% low EBP implementation, and 18.4% no EBP implementation. There were no significant predictors of EBP implementation (i.e., EBP implementation was not related to youth presenting problems, age, family adversity, therapist licensure status, therapist years of experience, language of treatment, or whether the parent was present in the session).

## Discussion

Routine care for adolescent ADHD tended to take adolescent-directed, rather than parent-teen collaborative or parent-directed approaches, and only seldom engaged additional stakeholders in session. Clinicians tended to integrate EBPs into most sessions (81.6% of work samples), but with very low extensiveness. The most frequently implemented practice elements were social skills training, skill application therapy homework, and time management skills training. Extensiveness of EBP implementation was not related to any predictors.

Evidence-based psychosocial treatments for adolescent ADHD typically employ a hybrid format that requires involvement of both parent and teen. Less than 30% of sessions utilized the parent-teen model and most delivered treatment to the adolescent alone (53.9%). Failure to engage the parent, or other stakeholders, in care may undermine the effectiveness of adolescent treatment (10, 11). Thus, a first strategic direction for community ADHD treatment is increasing engagement of stakeholders in sessions.

When EBPs were implemented, it was typically at a very low intensity (see Table 2). This finding echoes the broader community care literature (23–26). Fidelity to EBPs impacts intervention effectiveness across clinical contexts, including the treatment of externalizing disorders, like ADHD (27–30). Therefore, a second strategic direction for routine care is improving the integrity with which EBPs are delivered to adolescents with ADHD.

The most common focus of adolescent ADHD treatment in community contexts was social skills training (31.6% of sessions). However, meta-analysis demonstrates no effects for social skills training on adolescent ADHD (31). Over-implementation of this low-value element may undermine the effectiveness of routine psychosocial care for adolescents with ADHD. In contrast, common elements of adolescent ADHD EBPs were rarely implemented (9, 10, 32). Thus, a third strategic direction for the treatment of adolescent ADHD in routine care is: (1) increased emphasis on EBP common elements and (2) decreased emphasis on low value, traditionally implemented, elements such as social skills training.

We were unable to detect associations between EBP implementation and adolescent or provider characteristics. There was modest variability in extensiveness of EBP implementation, which may have prevented patterns from emerging. In addition, an inevitable limitation of community-based research is lower control and data collection rates than university trials. Thus, we were only able to collect usable work samples for about 2/3rds of the treated sample. Work samples were collected from four agencies in a single geographic region who primarily serve minority youth; it is unclear whether findings generalize to other contexts, such as non-Latinx families in other locations. It is also unclear if these findings would generalize to samples with higher medication utilization or higher rates of hyperactivity/impulsivity symptoms. It is unclear whether work samples adequately represent non-recorded sessions, which could introduce a selection bias.

Offering psychosocial treatment for adolescent ADHD (in addition to or instead of medication) is an opportunity to increase engagement in routine care services. However, to ensure effectiveness, care must reflect evidence-based practices. Based on this study, strategic directions for routine care include improving parent engagement in adolescent ADHD treatment, increasing the integrity with which EBPs are delivered, reducing low value practices (such as social skills training), and appropriately incorporating common elements of adolescent ADHD psychosocial treatment (9–11, 32). The work described herein might guide implementation and de-implementation efforts in routine care contexts, which may include provider workshops, dissemination of treatment manuals, and fidelity monitoring and feedback initiatives.

## Data availability statement

The datasets presented in this study can be found in online repositories. The names of the repository/repositories and accession number(s) can be found below: https://nda.nih.gov.

## Ethics statement

The studies involving human participants were reviewed and approved by Florida International University Institutional Review Board. Written informed consent to participate in this study was provided by the participants' legal guardian/next of kin.

## Author contributions

MS contributed to the trial administration, conceptualization, data analysis, and primary manuscript writing. JR contributed to data coding and manuscript editing. AR-D contributed to conceptualization, data coding, and manuscript editing. PG contributed to trial administration, conceptualization, and manuscript editing. All authors contributed to the article and approved the submitted version.

## Funding

This project was funded by grant R01 MH106587 from the National Institute of Mental Health (USA).

## Conflict of interest

MS receives book royalties from Guilford Press for a book on treating ADHD in adolescence using non-pharmacological approaches. The remaining authors declare that the research was conducted in the absence of any commercial or financial relationships that could be construed as a potential conflict of interest.

## Publisher's note

All claims expressed in this article are solely those of the authors and do not necessarily represent those of their affiliated organizations, or those of the publisher, the editors and the reviewers. Any product that may be evaluated in this article, or claim that may be made by its manufacturer, is not guaranteed or endorsed by the publisher.

## Footnotes

^1^In the calculation of inter-rater reliability, 12 codes had zero variance because they did not appear on any of the selected 20% of tapes. Inter-rater reliability therefore could not be calculated for these codes, but inter-rater agreement was 100%.
